# Synthesis of MAPA Reagents and 2-Alkyl(aryl)aminopyridines from 2-Bromopyridine Using the Goldberg Reaction

**DOI:** 10.3390/molecules27061833

**Published:** 2022-03-11

**Authors:** Daniel L. Comins

**Affiliations:** Department of Chemistry, North Carolina State University, Raleigh, NC 27695-8204, USA; dlcomins@ncsu.edu

**Keywords:** *N*-methyl-*N*-(2-pyridyl)formamide, 2-methylaminopyridine amides, 2-alkyl(aryl)aminopyridines, Goldberg reaction, copper-catalyzed reactions, cross-coupling reactions, 1,10-phenanthroline

## Abstract

A short and economical synthesis of various 2-methylaminopyidine amides (MAPA) from 2-bromopyridine has been developed using the catalytic Goldberg reaction. The effective catalyst was formed in situ by the reaction of CuI and 1,10-phenanthroline in a 1/1 ratio with a final loading of 0.5–3 mol%. The process affords high yields and can accommodate multigram-scale reactions. A modification of this method provides a new preparation of 2-*N*-substituted aminopyridines from various secondary *N*-alkyl(aryl)formamides and 2-bromopyridine. The intermediate aminopyridine formamide is cleaved in situ through methanolysis or hydrolysis to give 2-alkyl(aryl)aminopyridines in high yields.

## 1. Introduction

**Figure 1:**
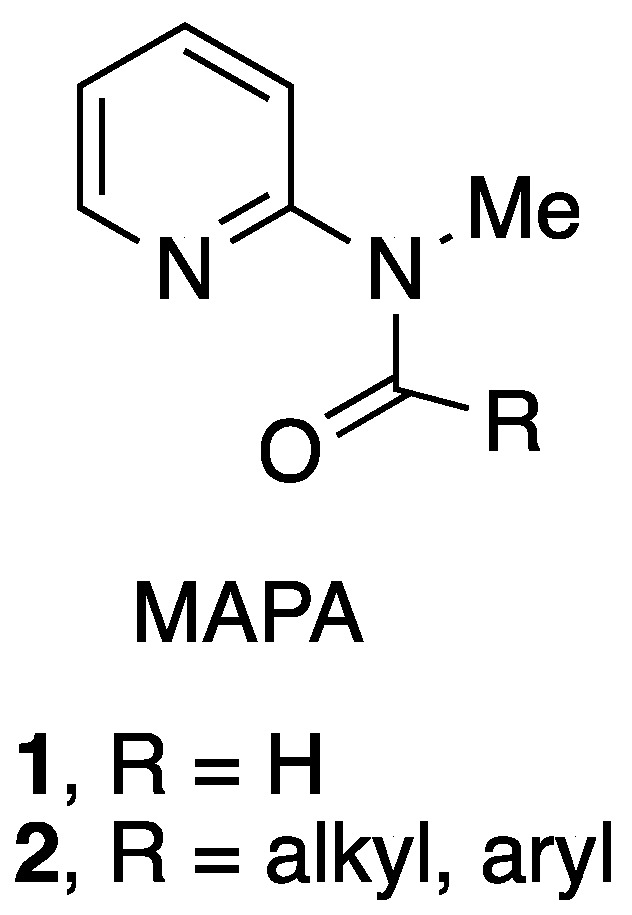
2-Methylaminopyridine amides.

The 2-methylaminopyridine formamide **1** and related amides **2** (MAPA) have been widely used as reagents for formylation and acylation of various organometallic reagents, amines, and other nucleophiles [1,2,3,4,5] (Figure 1).

The formamide **1** (MAPF), also known as the Comins–Meyers reagent [5], has been used in pharmaceutical and academic laboratories. MAPF and a few other MAPA reagents can be purchased commercially, although at present their cost is relatively high. The most common methods for preparing these amides are (1) formylation or acylation of 2-aminopyridine followed by methylation of the amide nitrogen [1], (2) formylation or acylation of 2-methylaminopyridine [2,3], and (3) treatment of pyridine *N*-oxide with chloroiminium ions [6]. The simplest and most direct of these procedures is (2); however, 2-methylaminopyridine is also costly at this time.

**Scheme 1:**
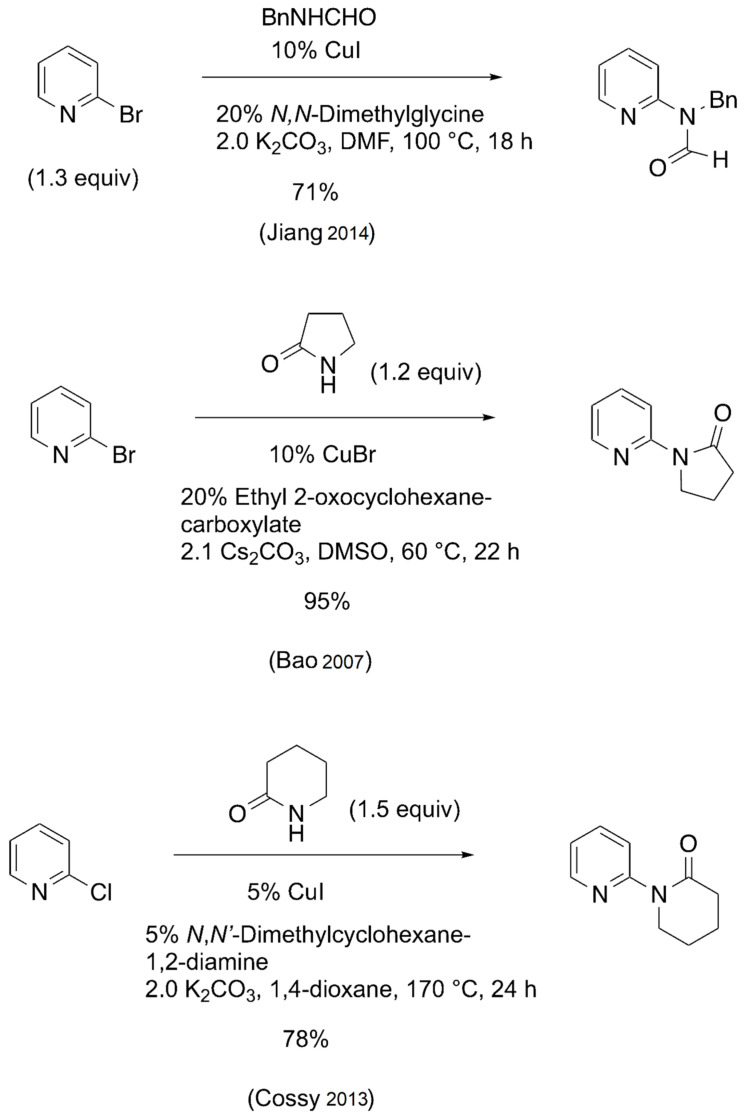
Goldberg reactions of 2-halopyridines with secondary amides and lactams [11,12,13].

A potentially inexpensive route to many MAPA-type compounds would be the direct conversion of 2-bromopyridine to the corresponding *N*-methyl amides via the Goldberg reaction. Although the catalytic Goldberg reaction has been extensively studied in recent years using various aryl and heteroaryl halides [7,8,9,10], only a few examples have been reported that provide MAPA-related compounds from 2-halopyridines. Some examples are depicted below (Scheme 1).

A practical large-scale conversion of 2-bromopyridine to MAPA reagents would require inexpensive ligands, copper salts, reagents, and solvents. In addition, low catalyst loading, low reagent and solvent toxicity, reasonable reaction temperature, and a simple workup would be desirable. Although many ligands have been found to promote the catalytic Goldberg reaction, diamine ligands appeared to be good candidates for the proposed study. Based on the work of Buchwald [14,15,16], Hosseinzadeh [17,18,19], and Ribas [20], 1,10-phenanthroline (phen) was chosen as the ligand for this project as it is inexpensive and has occasionally been found to be effective for some Goldberg reactions at low catalyst loading with a Cu/ligand ratio of 1/1.

## 2. Results and Discussion

### 2.1. Synthesis of MAPA Reagents ***1*** and ***2***

Initial studies began using 2-bromopyridine, *N*-methylformamide (NMF), CuI/phen (1/1), in *t*-AmOH or toluene. As indicated in Table 1, 1–2% of the catalyst was sufficient to give excellent yields of **1** in most cases. When *t*-AmOH was the solvent with potassium phosphate as the base (entry 1), the reaction proceeded well in 8 h; however, a significant amount of deformylation of the initial formamide product occurred, to give 2-methylaminopyridine (MAP) as a byproduct. Using 2% CuI/phen, toluene as the solvent, and K_2_CO_3_ with a small amount of K_3_PO_4_ as the base, MAPF (**1**) was afforded in high yields (entry 3). This reaction was also carried out on a 0.1 mol scale to give 11.5 g (85%) of MAPF, purified by distillation (entry 5). Preparation of the MAPA reagent *N*-methyl-*N*-(pyridin-2-yl)acetamide (**2a**) from inexpensive *N*-methylacetamide was examined next. Although the reaction times were longer than with NMF, similar results were obtained. As before, with *t*-AmOH as the solvent, the reaction proceeded, but partial deacylation occurred, giving some MAP as a byproduct (entry 6). The best conditions found used 5% *t*-AmOH/toluene as a solvent and K_3_PO_4_ as the base, to give an 88% isolated yield of MAPA **2a** (entry 8). To determine if benzamides could be made from 2-bromopyridine by this process, *N*-methylbenzamide was examined as the cross-coupling partner (entries 9–11). In this case, neat toluene was found to be the best solvent, providing the desired MAPA product **2b** in an 80% isolated yield (entry 11).

### 2.2. One-Pot Synthesis of 2-Alkyl(aryl)aminopyridines from 2-Bromopyridine and Secondary Formamides

Due to the electron-withdrawing nature of the pyridine ring, the acyl group of most 2-*N*-alkyl(aryl)aminopyridine amides is easily cleaved by hydrolysis or alcoholysis. This reactivity, observed in the above study when *t*-AmOH was the solvent (Table 1, entries 1, 6, and 9), encouraged us to investigate a one-pot conversion of 2-bromopyridine to 2-alkyl(aryl)aminopyridines by in situ methanolysis of the intermediate formamide product from the Goldberg reaction.

A practical multi-gram preparation of 2-methylaminopyridine (MAP) was the first goal. In this case, excess potassium phosphate was chosen as the base, as completion of the reaction in the first step to give the intermediate formamide (MAPF) is important. Furthermore, the excess base would help promote the cross-coupling, as well as the methanolysis in the second step. An incomplete reaction in the first step would complicate purification by distillation as 2-bromopyridine and MAP codistill. As shown in Table 2, entry 1, a mixture of NMF, 2-bromopyridine, 2% CuI/phen, and 2 equiv. K_3_PO_4_ in refluxing toluene for 12 h efficiently provided the intermediate MAPF in situ on a 0.1 mol scale. The toluene was removed under reduced pressure and replaced with methanol. Potassium carbonate (1 equiv.) was added, and the mixture was heated at reflux for 4 h to give a high yield (89%) of MAP after distillation. To determine the scope of this one-pot method, four other examples were carried out on a 10 mmol scale (Table 2, entries 2–5). Preparation of 2-(1-butylamino)pyridine (**3b**) was attempted next (entry 2). Using the general procedure, *N*-butylformamide and 2-bromopyridine gave a 90% isolated yield of **3b.** Using similar conditions (entry 3), *N*-benzylformamide provided an 89% yield of recrystallized 2-benzylaminopyridine (**3c**). The corresponding reaction with *N-tert*-butylformamide was proved to require some modification of the general procedure. The first step is the same, except the reaction time requires 48 h. This is undoubtedly due to the steric hindrance in the formamide cross-coupling partner. The intermediate 2-*tert*-butylaminopyridine formamide did not readily undergo methanolysis, but did hydrolyze with NaOH/EtOH at reflux, using the one-pot procedure in moderate yield. A higher yield and easier workup were obtained by first filtering the reaction mixture from the first step, prior to removal of the toluene. This two-pot procedure resulted in an 85% isolated yield of 2-*tert*-butylaminopyridine (**3d**). To determine if 2-arylaminopyridines could be made in one step by this methodology, *N-p*-tolylformamide and 2-bromopyridine were subjected to the one-pot reaction using a mixture of K_2_CO_3_ and K_3_PO_4_ as a base. The reaction times for both the first and second steps were relatively short, and the purified product **3e** was obtained in an 88% yield (entry 5).

Although there are now several methods that use alkyl- or arylamines in cross-coupling reactions with 2-halopyridines [21], many require an excess of the amine component or may not be amenable to large-scale synthesis. The method described here uses one equivalent of stable formamides that are commercially available or can be easily prepared at low cost. The simplicity of the process may also make it attractive as a candidate for large-scale synthesis of various 2-alkyl(aryl)aminopyridines.

### 2.3. Use of DMEDA as an Alternative Ligand for the above Goldberg Reactions

Although a general screening of ligands for the above studies was not carried out, the popular *N*,*N′*-dimethylethylenediamine (DMEDA) [7,8,9,10] was examined briefly to see how it competes with phen as a ligand for the Goldberg reaction of 2-bromopyridine and secondary amides. The usual loading for CuI/DMEDA reactions of this type is 1/2 with CuI at 5–10%. To perform a direct comparison with the reactions using phen, a loading of 2% CuI and 2–4% DMEDA was used. The cross-coupling with *N*-methylformamide (NMF) was examined first (Table 3, entries 1–3). Using CuI/DMEDA at 2%/4%, toluene as a solvent, and K_2_CO_3_/K_3_PO_4_ as a base provided an excellent yield of formamide **1** after 8 h at reflux (entry 1). Decreasing the amount of DMEDA ligand to 2% afforded a comparable result (entry 2). This is as good as what was obtained using phen as the ligand under similar conditions (Table 1, entries 2 and 3). When *N*-methylacetamide (NMA) was used as the amide coupling partner, poor results (8–10% yield) were obtained after 24 h reflux (Table 3, entries 4 and 5). This is in line with what Buchwald [16] reported on related reactions using CuI/DMEDA, NMA, and other aryl halides. Surprisingly, the more hindered *N*-methylbenzamide gave a good yield of amide **2b** after 36 h (Table 3, entry 6). The more reactive *N*-benzylformamide was examined next. Reactions using K_2_CO_3_ or K_3_PO_4_ as a base and CuI/DMEDA in a 2%/4% ratio were carried out. Both reactions worked well, providing the desired product in 80% and 84% yield, respectively (Table 3, entries 7 and 8). The hindered formamide, *t*-butylformamide, that worked well with phen as the ligand (Table 2, entry 4) was examined. Using a catalyst loading of 5% CuI and 10% DMEDA in toluene for 24 h at reflux, a low yield of the desired cross-coupling was observed (Table 3, entry 9).

## 3. Materials and Methods

### 3.1. General Information

The reagents used, unless stated otherwise, were purchased from commercial sources and used without further purification or drying: toluene (99%, ACROS, Geel, Belgium), *N*-methylacetamide (99%, TCI, Portland, OR, USA), *N*-formyl-*p*-toluidine (K&K, Saint Louis, MO, USA), copper(I) iodide (98%, Aldrich, Milwaukee, WI, USA), 1,10-phenanthroline monohydrate (reagent grade, Sigma, St. Louis, MO, USA)), anhydrous potassium phosphate, tribasic (97%, ACROS, Geel, Belgium), and potassium carbonate, anhydrous (99%, Alfa Aesar, Ward Hill, MA, USA). *N*-Methylformamide (99%, BTC, Cheadle, UK) and 2-bromopyridine (99%, AmBeed, Arlington Heights, IL, USA) were vacuum distilled and stored over 4A molecular sieves. *N*-Methylbenzamide [22], *N*-benzylformamide [23], *N*-butylformamide [24], and *N*-*tert*-butylformamide [25] were made according to procedures outlined in the literature. *Tert*-amyl alcohol (99%, Sigma-Aldrich, St. Louis, MO, USA) was dried over powdered 4A molecular sieves, distilled, and stored over 4A molecular sieves. Chromatographic purifications were carried out by radial PLC on a Chromatotron (Harrison Research, Palo Alto, CA, USA) using silica gel plates (Kieselgel 60 PF254). NMR spectra were obtained on a Bruker (Billerica, MA, USA) NEO 500 or 600 MHz spectrometer. All products are known compounds previously published in the literature. The ^1^H NMR spectra of the purified products are found in the Supplementary Materials.

### 3.2. Preparation of MAPA Compounds

#### 3.2.1. *N*-Methyl-*N*-(pyridin-2-yl)acetamide (**2a**)

General procedure A: to a 50 mL, one-necked, round-bottomed flask equipped with a reflux condenser and a magnetic stirring bar, 1,10-phenanthroline monohydrate (40 mg, 0.2 mmol), copper iodide (38 mg, 0.2 mmol), anhydrous potassium phosphate (4.25 g, 20 mmol), *N*-methylacetamide (731 mg, 10 mmol), 2-bromopyridine (0.95 mL, 10 mmol), and 5% *t*-AmOH/toluene (10 mL) were added. The mixture was stirred vigorously while the flask was flushed with nitrogen for 10 min. The mixture was then heated at reflux with stirring for 36 h. After cooling to rt, 20 mL of ethyl acetate was added, and the mixture was stirred at rt for 15 min. The mixture was vacuum-filtered through Celite with an ethyl acetate wash (2 × 15 mL). The filtrate was concentrated on a rotary evaporator to provide the crude product as a yellow oil (1.6 g), which was purified by radial PLC (silica gel, 10–50% acetone/hexanes/1% MeOH) to give 1.32 g (88%) of **2a** as a clear oil.

#### 3.2.2. *N*-Methyl-*N*-(pyridin-2-yl)benzamide (**2b**)

Following general procedure A, *N*-methylbenzamide, 2 mol% CuI/phen, K_3_PO_4_, and 2-bromopyridine in neat toluene provided 1.7 g (80%) of **2b** as a clear oil after purification by radial PLC (silica gel, 10–30% acetone/hexanes/1% MeOH).

#### 3.2.3. *N*-Methyl-*N*-(pyridin-2-yl)formamide (**1**)

Scale: 0.1 mol. To a 500 mL, one-necked, round-bottomed flask equipped with a reflux condenser and a magnetic stirring bar, 1,10-phenanthroline monohydrate (396 mg, 2.0 mmol), copper iodide (381 mg, 2.0 mmol), potassium carbonate (27.6 g, 0.2 mol), potassium phosphate (2.12 g, 10 mmol), *N*-methylformamide (5.84 mL, 0.1 mol), 2-bromopyridine (9.54 mL, 0.1 mol), and toluene (125 mL) were added. The mixture was stirred vigorously while the flask was flushed with nitrogen for 15 min. The mixture was then heated at reflux with stirring for 12 h. After cooling to rt, 100 mL of ethyl acetate was added, and the mixture was stirred at rt for 15 min. The mixture was vacuum-filtered through Celite with an ethyl acetate wash (2 × 75 mL). The filtrate was concentrated on a rotary evaporator to provide the crude compound as a yellow oil (13.5 g), which was purified by vacuum distillation to give 11.5 g (85%) of **1** as a clear oil, bp 95-100 °C (1.0 mmHg), lit. bp 71–72 °C (0.005 torr) [1].

### 3.3. Preparation of 2-Alkyl(aryl)aminopyridines

#### 3.3.1. *N*-Butylpyridin-2-amine (**3b**)

General procedure B: to a 50 mL, one-necked, round-bottomed flask equipped with a reflux condenser and a magnetic stirring bar, 1,10-phenanthroline monohydrate (40 mg, 0.2 mmol), copper iodide (38 mg, 0.2 mmol), potassium phosphate (4.25 g, 20 mmol), *N*-butylformamide (1.01 g, 10 mmol), 2-bromopyridine (0.95 mL, 10 mmol), and toluene (10 mL) were added. The mixture was stirred vigorously while the flask was flushed with nitrogen for 10 min. The mixture was then heated at reflux with stirring for 14 h. After cooling to rt, the toluene was removed on a rotary evaporator. Potassium carbonate (2 equiv., 2.8 g) and methanol (25 mL) were added, then the mixture was heated at reflux with stirring for 6 h. The methanol was removed on a rotary evaporator. Ethyl acetate (25 mL), 15% ammonium hydroxide (20 mL), and water (20 mL) were added to the flask. The mixture was vigorously stirred at rt for 15 min and then transferred to a 125 mL separatory funnel. The aqueous layer was extracted with ethyl acetate (2 × 15 mL). The combined organic extracts were washed with 10 mL portions of 15% Na_2_CO_3_, 50% brine, and brine, and then dried over K_2_CO_3_. Filtration through Celite and solvent removal in vacuo yielded the crude product as a yellow oil (1.5 g), which was purified by radial PLC (silica gel, 10–30% acetone/hexanes/1% MeOH) to give 1.35 g (90%) of **3b** as a low-melting white solid, mp 40–42 °C, lit. mp 40–41 °C [26].

#### 3.3.2. *N*-Benzylpyridin-2-amine (**3c**)

Following general procedure B, *N*-benzylformamide, 2 mol% CuI/phen, K_3_PO_4,_ and 2-bromopyridine in neat toluene (reflux 18 h) gave, after methanolysis with 1 equiv. of K_2_CO_3_ (reflux 4 h), 1.82 g of a white solid. Recrystallization from heptane/toluene afforded 1.64 g (89%) of **3c** as white crystals, mp 94–95 °C, lit. mp 94–96 °C [27].

#### 3.3.3. *N*-(p-Tolyl)pyridine-2-amine (**3e**)

Following general procedure B, *N*-*p*-tolylformamide, 1 mol% CuI/Phen, K_2_CO_3_/K_3_PO_4_, and 2-bromopyridine in neat toluene (15 mL, reflux 10 h) gave, after methanolysis (no K_2_CO_3_, reflux 3 h), 1.8 g of the crude product. Purification by radial PLC (silica gel, 20–30% acetone/hexanes) afforded 1.62 g (88%) of **3e** as a light tan solid, mp 105–106 °C, lit. mp 103–104 °C [28].

#### 3.3.4. *N*-(*tert*-Butyl)pyridine-2-amine (**3d**)

To a 50 mL, one-necked, round-bottomed flask equipped with a reflux condenser and a magnetic stirring bar, 1,10-phenanthroline monohydrate (59 mg, 0.3 mmol), copper iodide (57 mg, 0.3 mmol), potassium phosphate (4.25 g, 20 mmol), *N*-*tert*-butylformamide (1.01 g, 10 mmol), 2-bromopyridine (0.95 mL, 10 mmol), and 5% *t*-AmOH/toluene (10 mL) were added. The mixture was stirred vigorously while the flask was flushed with nitrogen for 10 min. The mixture was then heated at reflux with stirring for 48 h. After cooling to rt, 20 mL of ethyl acetate was added, and the mixture was stirred at rt for 15 min. The mixture was vacuum-filtered through Celite with an ethyl acetate wash (2 × 15 mL) into a 100 mL round-bottomed flask. The filtrate was concentrated on a rotary evaporator to yield 1.84 g of the crude intermediate as a yellow oil. Ethanol (40 mL) and 20% NaOH (10 mL) were added to the flask and the mixture was heated at reflux for 6 h (open condenser). Most of the EtOH was removed on a rotary evaporator. Methylene chloride (25 mL) and water (20 mL) were added, and the mixture was transferred to a separatory funnel. The aqueous layer was extracted with CH_2_Cl_2_ (2 × 15 mL). The combined organic layers were washed with 15 mL portions of 50% saturated brine and brine, dried over K_2_CO_3_, and concentrated to give 1.44 g of crude product. Purification by radial PLC (silica gel, 10–30% acetone/hexanes/1% MeOH) afforded 1.27 g (85%) of **3d** as a white solid, mp 52–53 °C, lit. mp 52–53 °C [29].

#### 3.3.5. *N*-Methylpyridin-2-amine (**3a**)

Scale: 0.1 mol. To a 500 mL, one-necked, round-bottomed flask equipped with a reflux condenser and a magnetic stirring bar, were added 1,10-phenanthroline monohydrate (396 mg, 2.0 mmol), copper iodide (381 mg, 2 mmol), potassium phosphate (42.5 g, 0.2 mol), *N*-methylformamide (6.5 g, 0.11 mol), 2-bromopyridine (15.8 g, 0.1 mol), and toluene (125 mL). The mixture was stirred vigorously while the flask was flushed with nitrogen for 15 min. The mixture was then heated at reflux with stirring for 12 h. After cooling to rt, the toluene was removed on a rotary evaporator. Potassium carbonate (1 equiv., 13.8 g) and methanol (125 mL) were added; then, the mixture was heated at reflux with stirring for 4 h. The methanol was removed on a rotary evaporator. Ethyl acetate (75 mL), 15% ammonium hydroxide (75 mL), and water (75 mL) were added to the flask. The mixture was vigorously stirred at rt for 20 min and then transferred to a 500 mL separatory funnel. The aqueous layer was extracted with ethyl acetate (3 × 25 mL). The combined organic extracts were washed with 25 mL portions of 15% Na_2_CO_3_, 50% brine, and brine, and then dried over K_2_CO_3_. Filtration through Celite and solvent removal in vacuo provided the crude product as a yellow oil (10.6 g), which was purified by vacuum distillation to give 9.4 g (89%) of **3a** as a clear oil, bp 93–96 °C (28 mmHg), lit. bp 100–102 °C (18 mmHg) [30].

### 3.4. Preparation of MAPA Compounds from 2-Bromopyridine Using DMEDA as Ligand

All reactions described in Table 3 were carried out on a 10 mmol scale using general procedure A with DMEDA replacing phen as the ligand. The yields were determined by ^1^H NMR using 1, 3, 5-trimethylbenzene as a standard. All the products are known compounds and were not purified.

## 4. Conclusions

A general and economical method for the synthesis of various 2-methylaminopyridine amides (MAPA) from 2-bromopyridine has been developed. The catalytic Goldberg reaction using copper iodide and 1,10-phenanthroline as a catalyst converts 2-bromopyridine and secondary *N*-methylamides into the desired MAPA compounds in excellent yields. Although DMEDA works well as the ligand in some of the reactions studied, phen is more effective when a sterically hindered or less reactive secondary amide is the coupling partner. A modification of this process using an *N*-alkyl(aryl)formamide as a starting material provides a one-pot synthesis of 2-alkyl(aryl)aminopyridines from 2-bromopyridine. The intermediate aminopyridine formamide is cleaved via in situ methanolysis or hydrolysis to afford the *N*-substituted 2-aminopyridine products in high yields. Both of these methods can conveniently be carried out on a multi-gram scale.

## Figures and Tables

**Table 1:** Goldberg reactions of 2-bromopyridine and secondary amides.

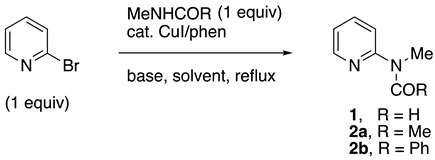						
Entry ^a^	Amide (R)	CuI/phen (mol%) ^b^	Solvent	Base (Equiv.)	Time ^c^ (h)	Yield ^d^ (%)
1	H	2	*t*-AmOH	K_3_PO_4_ (2)	8	**1**, 65 (28) ^e^
2	H	2	PhMe/ *t*-AmOH (9:1)	K_2_CO_3_ (1.3)	8	**1**, 93
				K_3_PO_4_ (0.1)		
3	H	1	PhMe	K_2_CO_3_ (1.3)	8	**1**, 92
				K_3_PO_4_ (0.05)		
4	H	0.5	PhMe	K_2_CO_3_ (1.3)	8	**1**, 88
5	H	2	PhMe	K_2_CO_3_ (2)	12	**1**, 85 ^f^
				K_3_PO_4_ (0.1)		
6	Me	2	*t*-AmOH	K_3_PO_4_ (2)	24	**2a**, 75 (10) ^e^
7	Me	2	PhMe/ *t*-AmOH (9:1)	K_3_PO_4_ (2)	24	**2a**, 84
8	Me	2	5% *t*-AmOH/ PhMe	K_3_PO_4_ (2)	36	**2a**, 92 (88) ^g^
9	Ph	2	*t*-AmOH	K_3_PO_4_ (2)	36	**2b**, 68 (11)^e^
10	Ph	2	PhMe/ *t*-AmOH (9:1)	K_3_PO_4_ (2)	36	**2b**, 85
11	Ph	2	PhMe	K_3_PO_4_ (2)	36	**2b**, 88 (80) ^g^

^a^ Reactions were generally performed on a 10 mmol scale. ^b^ The ratio of CuI/phen was 1/1. ^c^ Time at reflux. ^d^ Yield determined by ^1^H NMR using 1,3,5-trimethylbenzene as a standard. ^e^ NMR yield of hydrolyzed product (2-methylaminopyridine) formed in situ. ^f^ Yield of product purified by distillation; reaction scale was 0.1 mol. ^g^ Yield of product purified by chromatography.

**Table 2:** One-pot Synthesis of 2-Alkyl(aryl)aminopyridines from 2-Bromopyridine and Secondary Formamides.

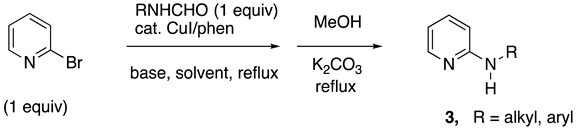						
Entry ^a^	Formamide R	CuI/phen (mol%) ^b^	Solvent, Base; 1st Step	Time ^c^ (h) 1st Step	Time ^c^ (h) 2nd Step	Yield ^d^ (%)
1	Me	2	PhMe	12	4	**3a**, 89 ^e^
			K_3_PO_4_ (2)			
2	Bu	2	PhMe	14	6	**3b**, 90 ^f^
			K_3_PO_4_ (2)			
3	Bn	2	PhMe	18	4	**3c**, 89 ^g^
			K_3_PO_4_ (2)			
4	*t*-Bu	3	5% *t*-AmOH/ PhMe	48 ^h^	6 ^i^	**3d**, 85 ^f^
			K_3_PO_4_ (2)			
5	*p*-tol	1	PhMe	10	3	**3e**, 88 ^f^
			K_2_CO_3_ (1.5)			
			K_3_PO_4_ (0.5)			

^a^ Reactions were generally performed on a 10 mmol scale. ^b^ The ratio of CuI/phen was 1/1. ^c^ Time at reflux. ^d^ Isolated yields. ^e^ Reaction scale was 0.1 mol and product was purified by distillation. ^f^ Product was purified by chromatography. ^g^ Product was purified by recrystallization. ^h^ The product mixture was filtered prior to toluene removal. ^i^ EtOH and 20% NaOH were used in the 2nd step.

**Table 3:** Reactions of 2-bromopyridine and secondary amides using DMEDA as the ligand.

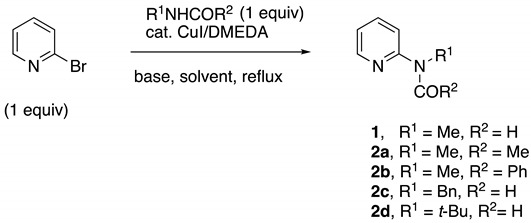						
Entry ^a^	Amide R^1^, R^2^	CuI/DMEDA mol%, Ratio	Solvent	Base (Equiv.)	Time ^b^ (h)	Yield ^c^ (%)
1	Me, H	2/4	PhMe	K_2_CO_3_ (1.3)	8	**1**, 92
				K_3_PO_4_ (0.1)		
2	Me, H	2/2	PhMe	K_2_CO_3_ (1.3)	8	**1**, 91
				K_3_PO_4_ (0.1)		
3	Me, H	2 (no ligand)	PhMe	K_2_CO_3_ (2)	8	**1**, 16
				K_3_PO_4_ (0.1)		
4	Me, Me	2/4	PhMe	K_3_PO_4_ (2)	24	**2a**, 8
5	Me, Me	2/2	5% *t*-AmOH/ PhMe	K_3_PO_4_ (2)	24	**2a**, 10
6	Me, Ph	5/10	PhMe	K_3_PO_4_ (2)	36	**2b**, 81
7	Bn, H	2/4	PhMe	K_2_CO_3_ (2)	18	**2c**, 80
8	Bn, H	2/4	PhMe	K_3_PO_4_ (2)	18	**2c**, 84
9	t-Bu, H	5/10	5% *t*-AmOH/ PhMe	K_3_PO_4_ (2)	24	**2d**, 12

^a^ Reactions were generally performed on a 10 mmol scale. ^b^ Time at reflux. ^c^ Yield determined by ^1^H NMR using 1,3,5-trimethylbenzene as a standard.

## Data Availability

The data presented in this study are available in this article and Supplementary Materials.

## Supplementary Materials

The following supporting information can be downloaded at: https://www.mdpi.com/article/10.3390/molecules27061833/s1. Figures S1–S8: Copies of ^1^H NMR spectra of all purified compounds (**1**, **2a**, **2b**, **3a**–**e**). References [31,32,33] are cited in the Supplementary Materials.
