# Supplementary material for: Synthesis of MAPA Reagents and 2-Alkyl(aryl)aminopyridines from 2-Bromopyridine Using the Goldberg Reaction
Source: Molecules. 2022 Mar 11;27(6):1833. doi: 10.3390/molecules27061833 (PMC8952803; doi:10.3390/molecules27061833)
Supplement: Supplementary file 1 [file molecules-27-01833-s001.zip › molecules-1623141-supplementary.pdf]

Supplementary Materials

Synthesis of MAPA Reagents and 2-Alkyl(aryl)aminopyridines from 2-Bromopyridine using the Goldberg Reaction

Daniel L. Comins [1,*]

[1]Department of Chemistry, North Carolina State University, Raleigh, North Carolina 27695-8204; dlcomins@ncsu.edu

Table of Contents

^{1}H NMR spectra of all purified products (**1**, **2a-b**, **3a-e**). S2-S9

All products are known compounds previously published in the literature. Spectral data and properties of the products are in agreement with literature values. The ^{1}H NMR spectra of the purified products are found below.

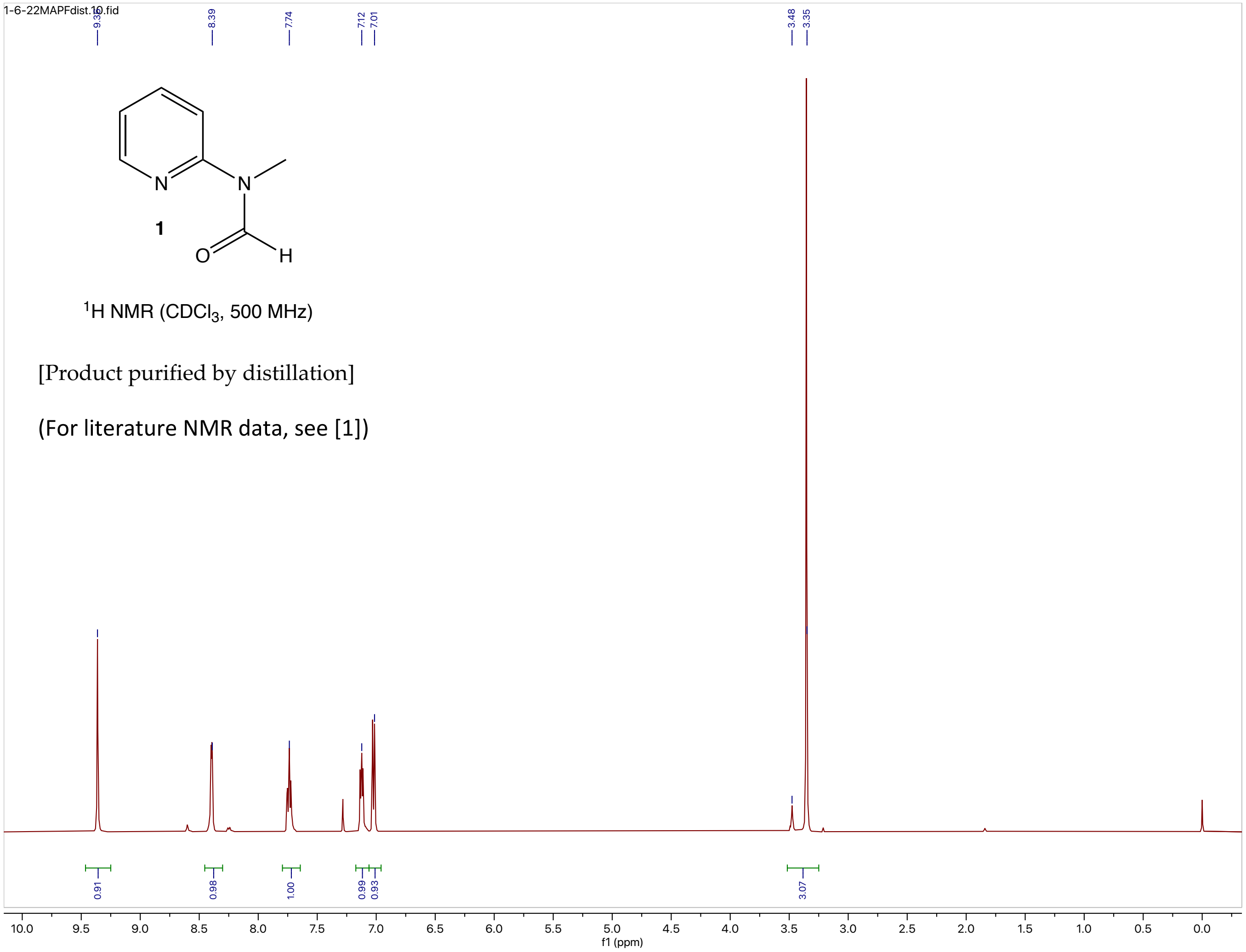

Figure S1. ^{1}H NMR spectrum of **1.**

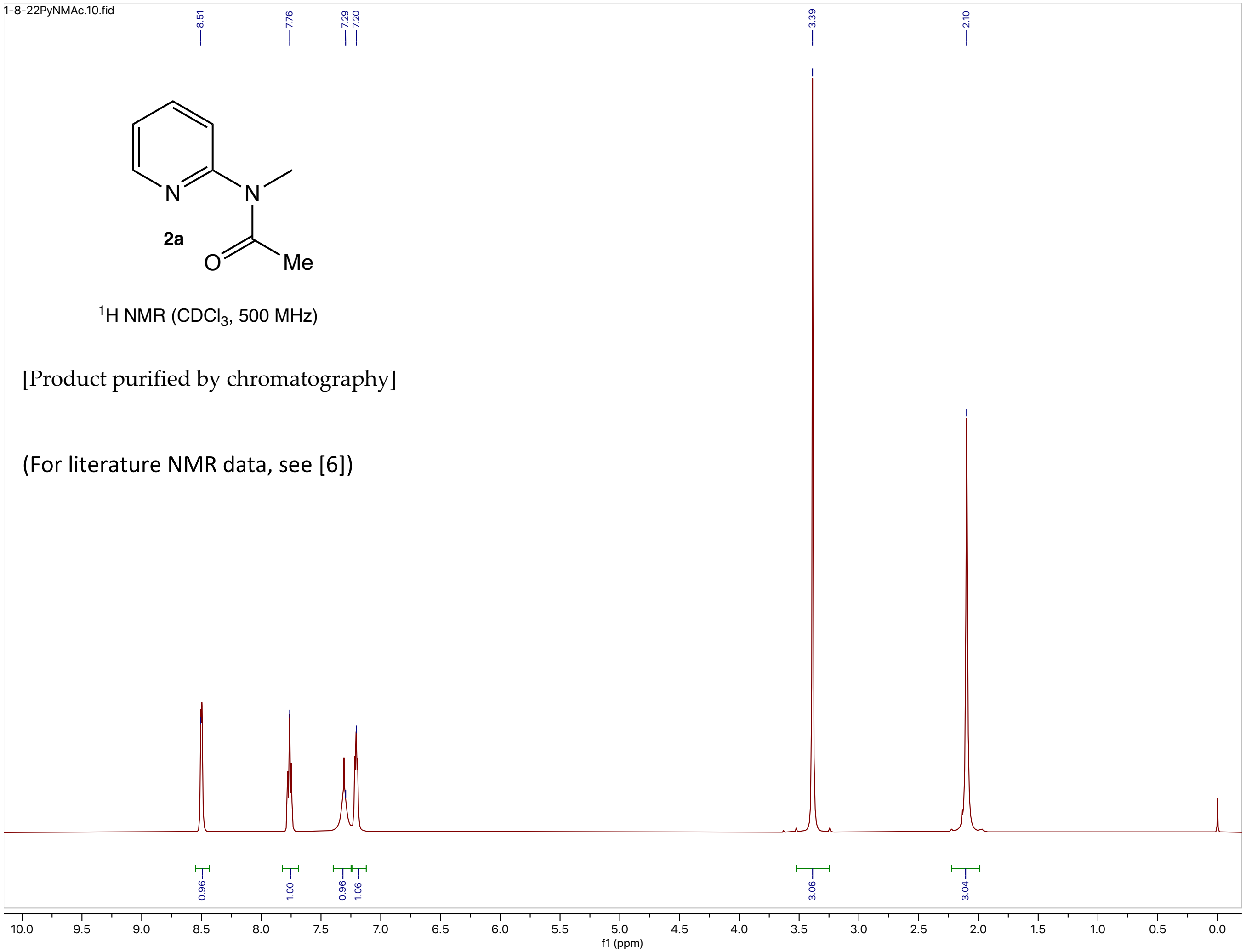

Figure S2. ^{1}H NMR spectrum of **2a.**

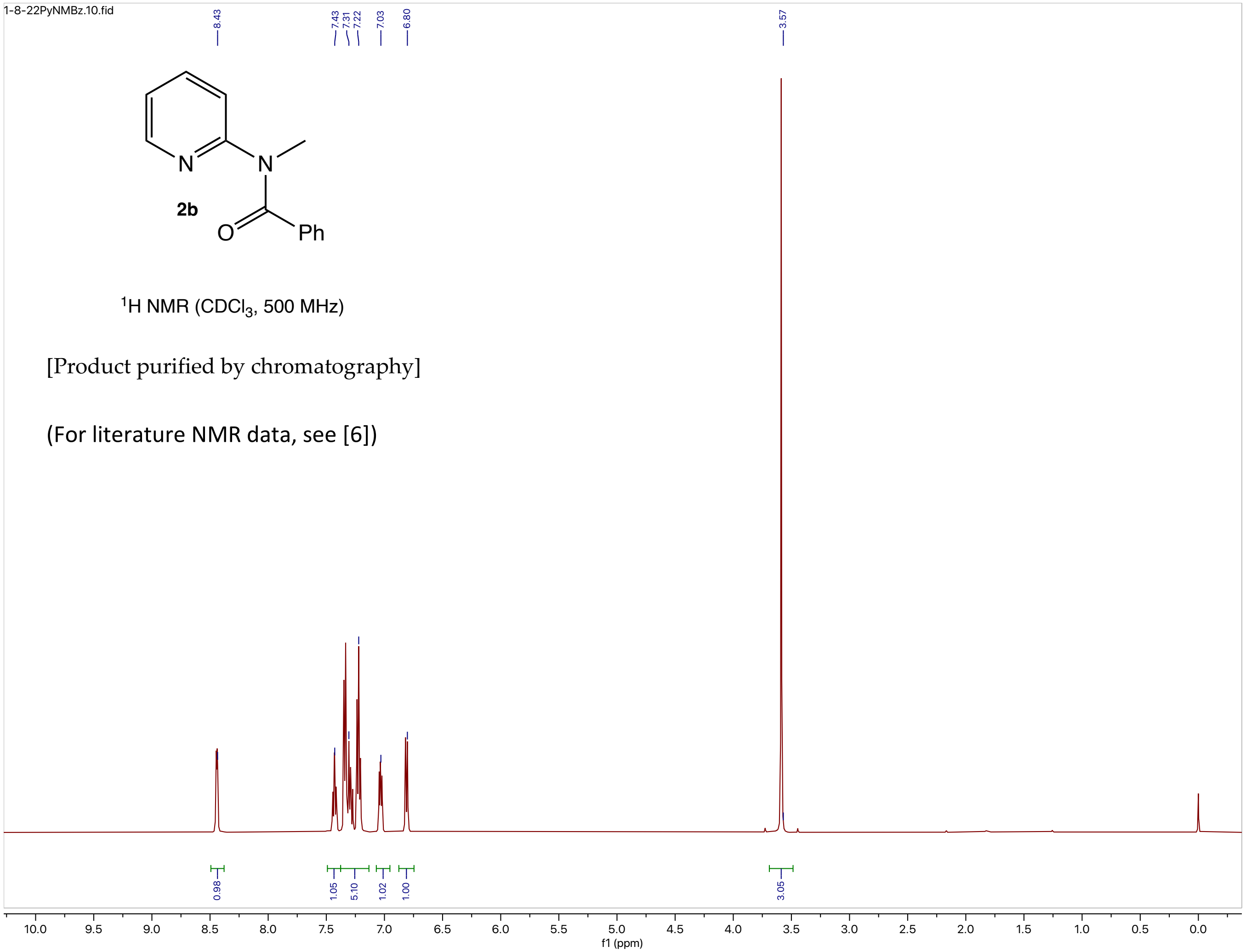

Figure S3. ^{1}H NMR spectrum of **2b.**

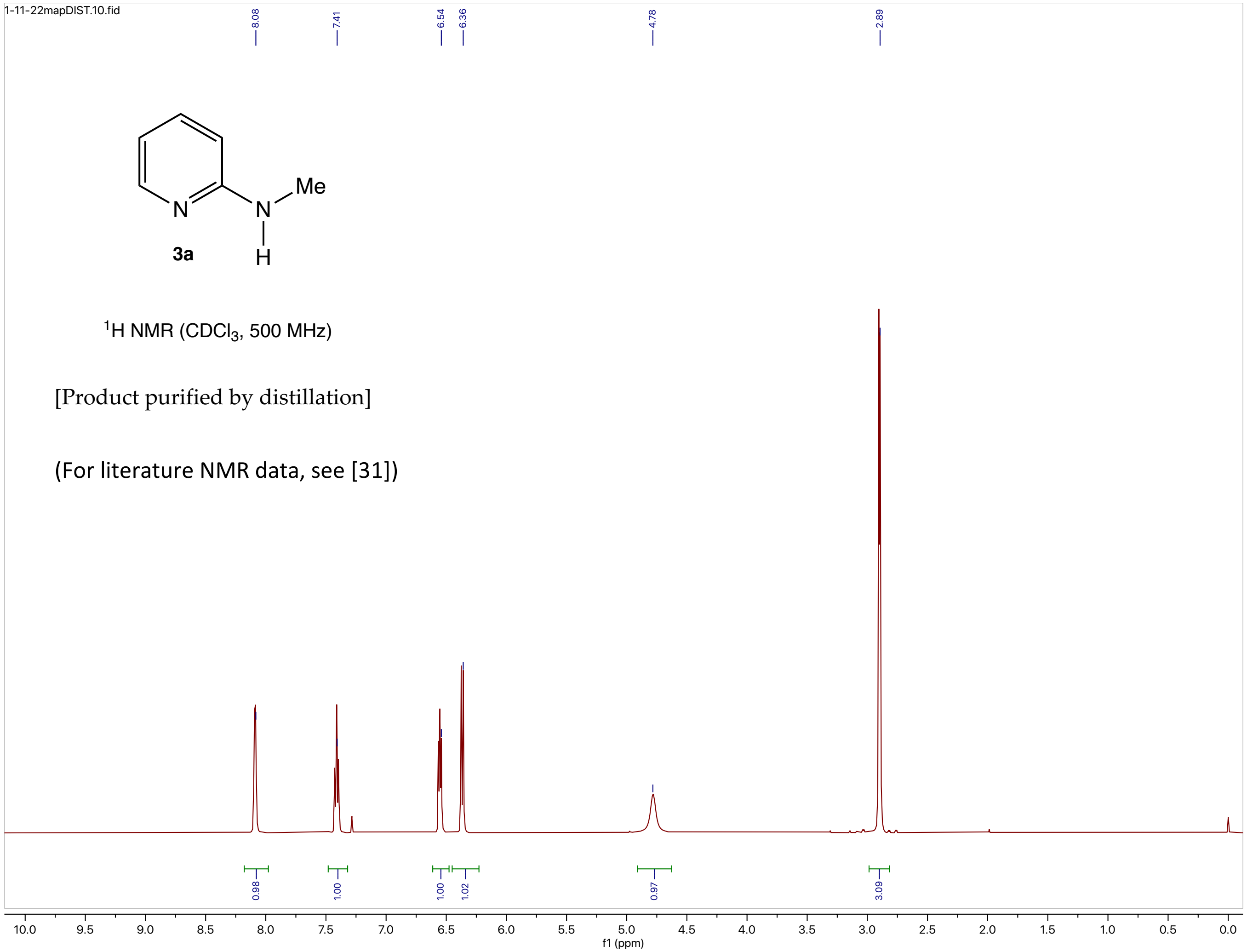

Figure S4. ^{1}H NMR spectrum of **3a.**

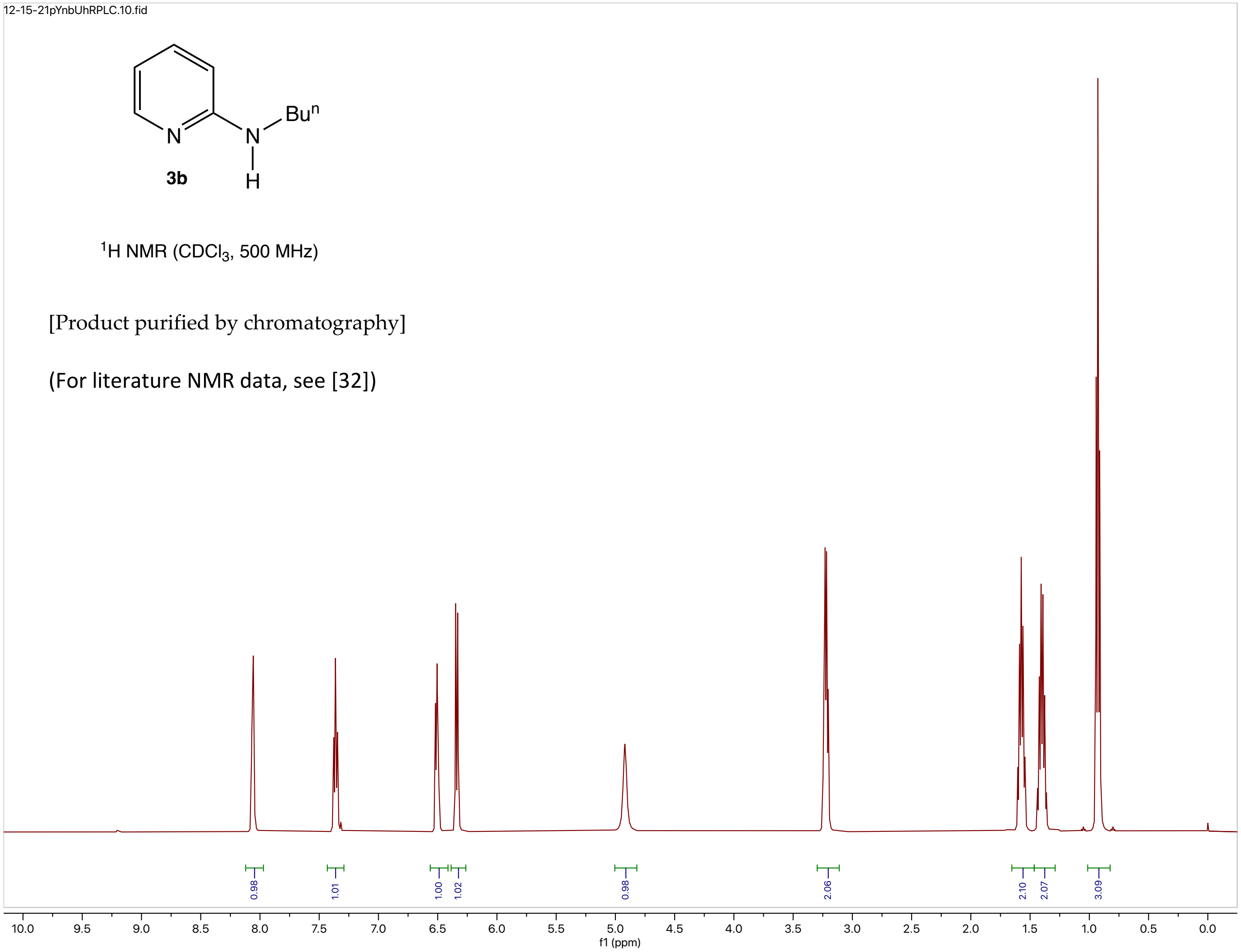

Figure S5. ^{1}H NMR spectrum of **3b**.

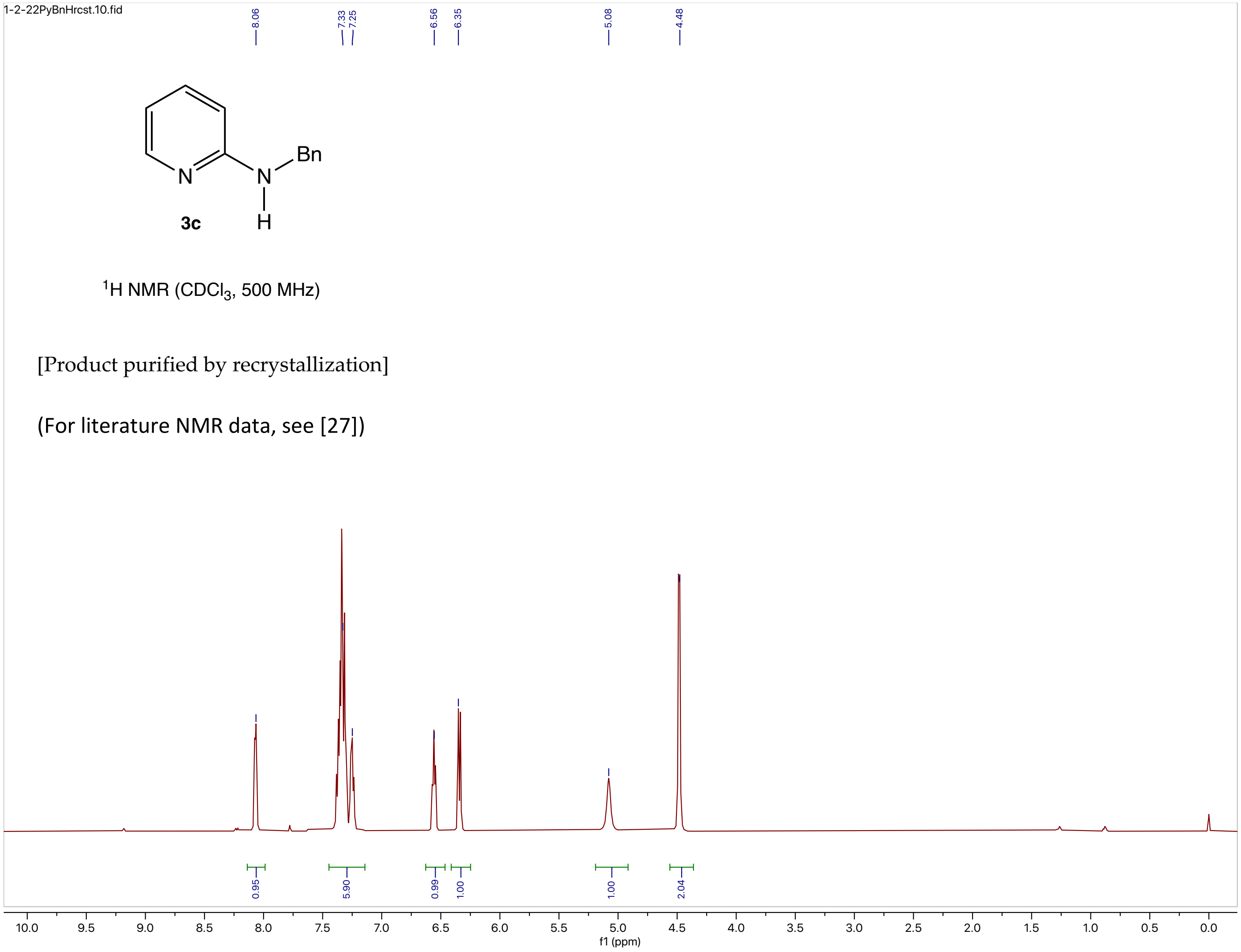

Figure S6. ^{1}H NMR spectrum of **3c.**

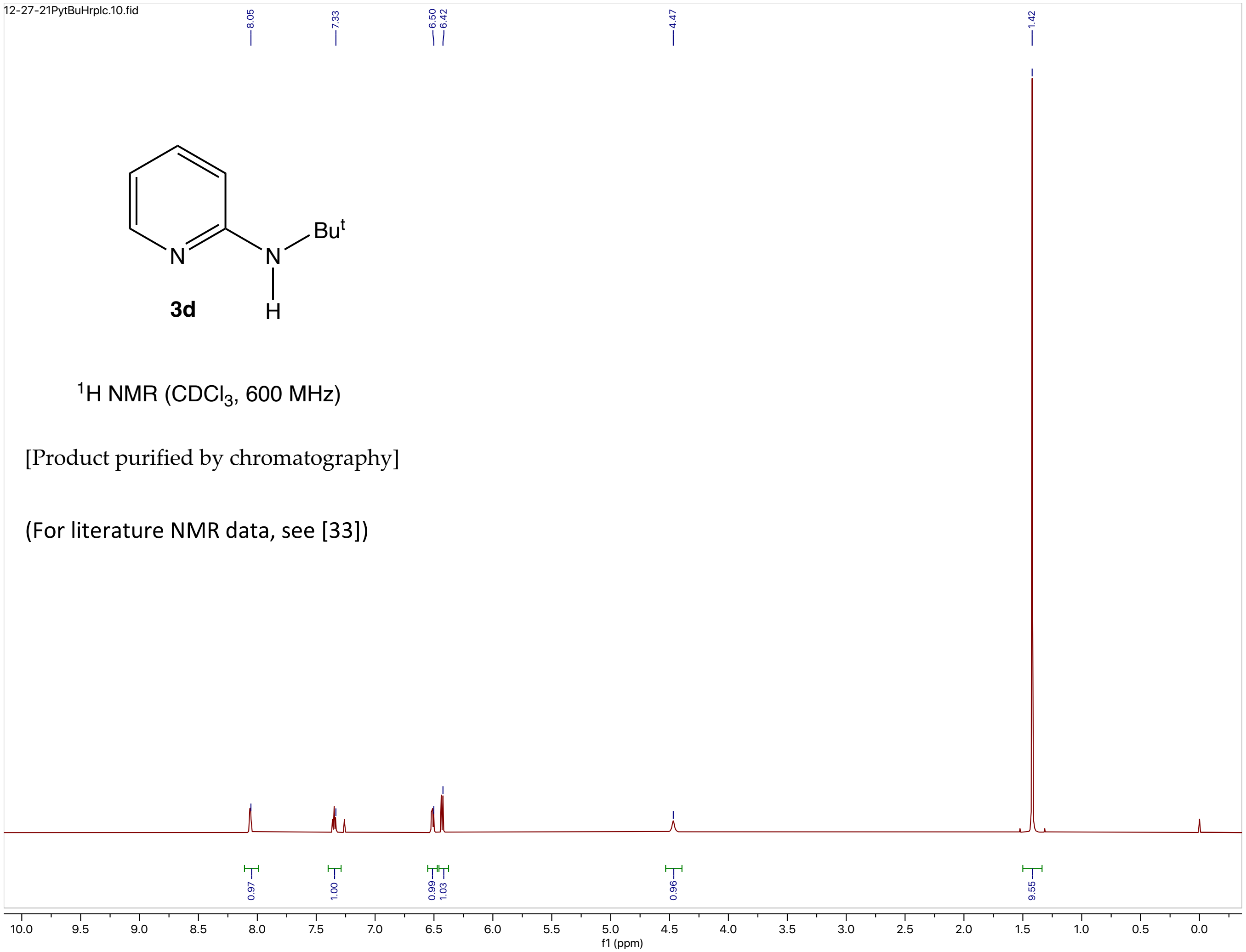

Figure S7. ^{1}H NMR spectrum of **3d.**

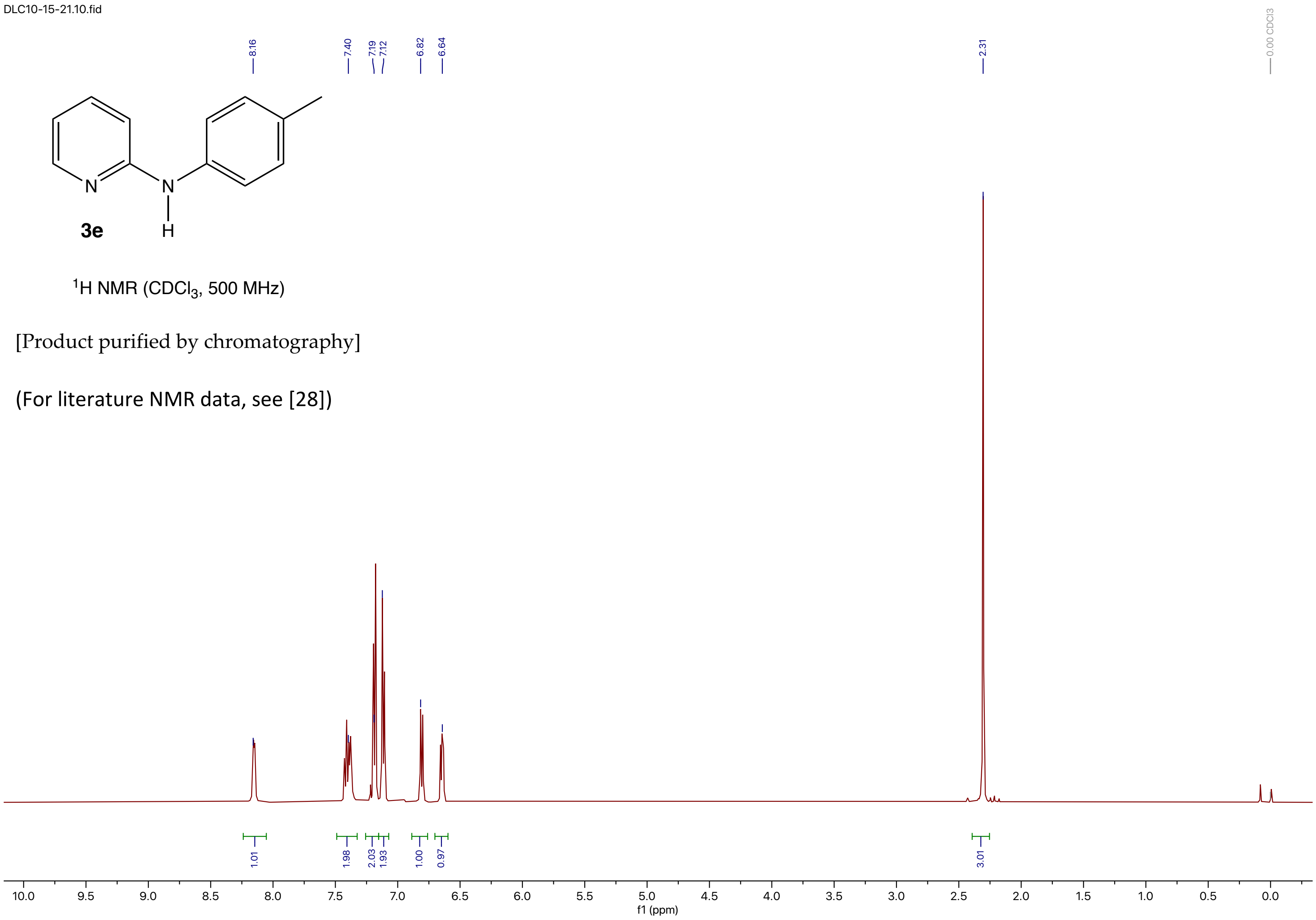

Figure S8. ^{1}H NMR spectrum of **3e.**
